# Constituent-Material-Anchored Continual Learning for Full Stress–Strain Prediction of Multi-Material PETG/PC-ABS MEX Laminates

**DOI:** 10.3390/polym18131573

**Published:** 2026-06-24

**Authors:** Ramachandran Avala Subramanian, Mahalingam Nainaragaram Ramasamy, Michal Prauzek, Quoc-Phu Ma, Jaromir Konecny, Ales Sliva

**Affiliations:** 1 Faculty of Electrical Engineering and Computer Science, VSB–Technical University of Ostrava, 17. Listopadu 15/2172, 70800 Ostrava, Czech Republic; ramachandran.avala.subramanian@vsb.cz (R.A.S.); jaromir.konecny@vsb.cz (J.K.); 2 Faculty of Mechanical Engineering, VSB–Technical University of Ostrava, 17. Listopadu 15/2172, 70800 Ostrava, Czech Republic; mahalingam.nainaragaram.ramasamy@vsb.cz (M.N.R.); phu.ma.quoc@vsb.cz (Q.-P.M.); ales.sliva@vsb.cz (A.S.)

**Keywords:** material extrusion, PETG/PC-ABS laminates, full stress–strain prediction, continual learning, constituent-material anchors, rule of mixtures

## Abstract

Predicting the tensile response of multi-material parts produced by material extrusion (MEX) remains difficult because the final behavior depends on both the constituent polymers and the quality and arrangement of dissimilar interfaces. This study introduces a constituent-material-anchored, phase-aware continual-learning framework for full stress–strain curve prediction of PETG/PC-ABS laminate coupons. Experimentally measured PETG and PC-ABS reference curves were combined through a rule-of-mixtures baseline; an XGBoost residual model then learned pointwise corrections using strain, baseline stress, mechanical phase label, and PETG thickness fraction as inputs. Validation used five PETG reference coupons, five PC-ABS reference coupons, five C1 laminate coupons, two C2 out-of-distribution coupons, and three coupons for each model-suggested Rank 1–3 architecture. UTS agreement alone was not sufficient: Rank 2 had a zero-shot UTS error of only −0.18% but a full-curve RMSE of 20.74%. After the first architecture-specific coupon was introduced, RMSE decreased from 12.34% to 2.72% for C1, from 18.60% to 6.38% for C2, from 21.04% to 6.93% for Rank 1, from 20.74% to 7.50% for Rank 2, and from 19.40% to 7.48% for Rank 3. The framework therefore provides a data-efficient, interpretable proof of concept for laminate screening and tensile-curve prediction, while its broader statistical robustness and extension to other loading modes require larger datasets.

## 1. Introduction

Material extrusion (MEX), including fused filament fabrication (FFF) and fused deposition modeling (FDM), is widely used for polymer additive manufacturing because it enables low-cost fabrication of complex thermoplastic parts with relatively simple equipment. Its mechanical performance is nevertheless difficult to predict because printed coupons are affected by process-sensitive variables such as layer height, raster orientation, nozzle temperature, print speed, thermal history, porosity, inter-road fusion, and interlayer bonding. These factors can produce anisotropy, weak interlayer adhesion, void formation, and stress–strain responses that differ from conventionally manufactured materials [1,2,3,4].

The prediction problem becomes more demanding in multi-material MEX. In addition to the process effects present in single-material prints, a laminate made from dissimilar polymers is governed by thermal compatibility, wetting, interdiffusion, load transfer across material transitions, and the number and location of interfaces. Consequently, the tensile curve of a PETG/PC-ABS laminate cannot be assumed to be a simple average of the two constituent responses. This material pair was selected because PETG and PC-ABS combine different stiffness, ductility, thermal resistance, and impact-related characteristics, and because earlier characterization of PETG/PC-ABS MEX laminates showed measurable tensile penalties relative to ideal constituent-material load sharing [5,6,7].

Machine learning has increasingly been used to reduce experimental trial-and-error in MEX and related polymer additive-manufacturing problems. Review studies identify data-driven models as useful tools for nonlinear process–property relationships [1,2,3]. Earlier tensile-property studies used group-method-of-data-handling models, artificial neural networks, adaptive neuro-fuzzy inference systems, fuzzy logic, and hybrid optimization strategies to predict tensile strength or optimize printing parameters [8,9,10,11,12,13,14,15,16]. More recent work has adopted deep learning, Gaussian-process regression, random forests, boosting, support-vector methods, and ensemble learning for tensile or related mechanical-property prediction [17,18,19,20,21,22,23,24,25,26,27,28].

Recent studies have also expanded mechanical-capacity prediction beyond standard polymer-MEX tensile benchmarks, including PETG-specific models, multi-material ABS/PETG optimization, reinforced or fiber-filled polymers, and broader data-driven capacity prediction in lattice structures, castings, UHPC-strengthened columns, UHPC compressive strength, and additively manufactured Ti-6Al-4V fatigue strength [29,30,31,32,33,34,35,36,37,38,39,40,41,42,43,44,45,46,47]. These studies confirm the value of data-driven capacity prediction, but most remain focused on scalar outputs such as strength, modulus, fatigue strength, or load capacity rather than the full tensile trajectory.

Three limitations motivate the present work. First, scalar endpoints such as UTS or elongation do not fully describe stiffness evolution, nonlinear transition, peak-stress development, or pre-failure curve shape. Second, many studies validate by internal train–test splitting within a known design space; this is not equivalent to testing genuinely unseen laminate architectures. Third, most models do not explicitly represent different mechanical regimes along a tensile curve, even though elastic, hardening, UTS-neighborhood, and post-UTS regions can have different error characteristics. These limitations are especially important for interface-sensitive multi-material laminates, where two stacks with similar strength may still have different stress–strain trajectories and different design implications.

The principal methodological novelty of this study is the combination of four elements in one experimentally validated workflow: (i) constituent-material reference curves are used as physically interpretable anchors; (ii) a rule-of-mixtures (RoM) baseline is corrected by a learned pointwise residual rather than replaced by a purely black-box model; (iii) the residual model is phase-aware and is trained on an adaptive strain grid refined near yield, high-curvature, and UTS regions; and (iv) the model is updated sequentially as laminate coupons become available and is evaluated on unseen architectures. In the present implementation, the residual regressor uses strain, RoM stress, phase label, and PETG thickness fraction as inputs. The method is therefore designed for data-efficient tensile-curve learning, not for claiming universal generalization across all polymer systems.

Table 1 summarizes the position of the present work relative to representative machine-learning-based mechanical prediction studies.

Accordingly, this study addresses the following research questions:

1. To what extent can constituent-material tensile anchors predict the full tensile stress–strain curve of PETG/PC-ABS laminates fabricated by MEX?
2. How much does phase-aware residual correction improve full-curve prediction relative to the initial RoM baseline, particularly near mechanically important regions such as yield, high-curvature transitions, and UTS?
3. Can the workflow support out-of-distribution laminate screening by predicting previously unseen PETG/PC-ABS architectures that are subsequently fabricated and tested experimentally?

## 2. Materials and Methods

This study followed a constituent-material-anchored, physics-informed, phase-aware residual-learning workflow for predicting the full tensile stress–strain response of PETG/PC-ABS laminates fabricated by material extrusion. The methodology was organized in sequential stages: experimental acquisition of pure PETG and pure PC-ABS reference curves, construction of constituent-material anchor curves, generation of an initial RoM baseline, preprocessing and quality checking of laminate coupon data, mechanical phase identification, adaptive strain-grid refinement, residual correction using a trained regression model, continual model updating, and out-of-distribution (OOD) laminate screening. Pure PETG and pure PC-ABS coupons were used only as single-material reference inputs for constructing the baseline response, while laminate coupons were used to learn the deviation between the RoM prediction and the measured full tensile curve. The workflow was designed as a data-efficient experimental strategy for the present PETG/PC-ABS system, not as a claim of universal robustness across all polymer pairs or loading modes.

**Figure 1:**
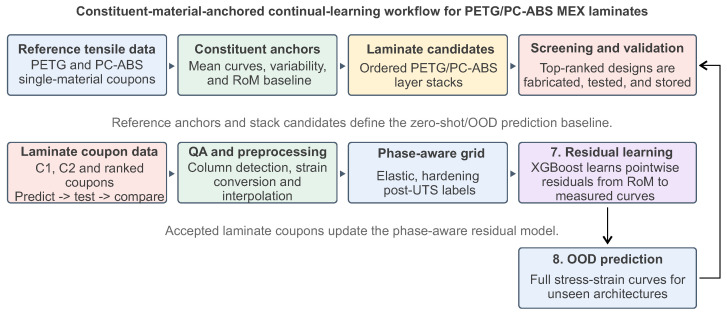
Methodological workflow with enlarged labels. The workflow links single-material reference tensile curves, constituent-anchor construction, laminate-coupon preprocessing, phase-aware residual learning, OOD prediction, and model-guided experimental validation.

Figure 1 summarizes the complete methodological workflow used in this study. The process begins with tensile testing of pure PETG and pure PC-ABS constituent materials, which provide the experimental reference curves for the constituent-anchor construction. These constituent anchors are then combined using a material-fraction-based rule-of-mixtures formulation to generate an initial physics-informed OOD prediction for PETG/PC-ABS laminate architectures.

In parallel, experimentally tested laminate coupons are acquired and passed through quality assurance and preprocessing, including strain standardization, interpolation, curve cleaning, and consistency checks. The processed curves are then segmented into mechanical phases, including elastic, hardening, and post-UTS/necking regions, and the strain grid is adaptively refined near mechanically important regions. The residual error between the measured laminate response and the rule-of-mixtures baseline is calculated pointwise and used to train the phase-aware residual-learning model. The trained model corrects the initial baseline prediction and produces full stress–strain curve predictions for unseen laminate architectures. These predictions are then evaluated, used for design screening, and ranked according to the implemented selection score. The top-ranked laminate designs are finally selected for experimental validation, and the newly obtained coupon data can be added back into the workflow for sequential model updating.

### 2.1. Definition of OOD Laminate Validation

In this study, an OOD laminate is formally defined at the architecture level. A prediction is considered OOD when the ordered PETG/PC-ABS layer stack being predicted has not contributed any coupon to the residual-learning memory before that prediction is made. This definition differs from a random train–test split of points from the same curve or coupons from the same architecture. Laminate C2 and the Rank 1–3 architectures therefore represent OOD cases at the time of their first prediction because their stack sequences were unseen by the correction model. After a coupon from a given architecture is accepted into memory, later predictions for that same architecture are described as architecture-specific updates rather than purely blind OOD predictions.

### 2.2. Materials, Coupon Fabrication, and Tensile Testing

This section defines the experimental material system and coupon sets used to support the constituent-anchor and incremental-learning workflow. Pure PETG and pure PC-ABS coupons were used as single-material reference coupons for constructing the reference tensile curves, whereas PETG/PC-ABS laminate coupons were treated as laminate architectures for zero-shot comparison, out-of-distribution validation, model-guided design assessment, and subsequent incremental updating. The material grades, coupon geometry, fabrication settings, and tensile testing protocol were based on the previously reported PETG/PC-ABS material extrusion study, while the present work reorganizes these experimental inputs for full-curve prediction and residual-learning methodology [5,6,7].

#### 2.2.1. Materials

Two commercial thermoplastic filaments supplied by Fiberlogy, Brzezie, Poland, were used as the constituent materials: Fiberlogy EASY PET-G and Fiberlogy PC/ABS. Both materials were used with a nominal filament diameter of 1.75 mm. The Fiberlogy EASY PET-G product line is described by the supplier as a glycol-modified PET material intended for mechanically and chemically resistant printed parts, whereas the Fiberlogy PC/ABS product line combines polycarbonate with ABS for improved impact resistance, chemical resistance, and thermal stability [5,6].

Pure PETG and pure PC-ABS coupons were fabricated and tested separately before the laminate-learning workflow. Five repeated tensile coupons were used for each constituent material,

(1) $$n_{\mathrm{PETG}}=5,\;n_{\mathrm{PC}-\mathrm{ABS}}=5,$$

where $n_{\mathrm{PETG}}$ and $n_{\mathrm{PC}-\mathrm{ABS}}$ denote the number of valid single-material reference coupons used for PETG and PC-ABS, respectively. These single-material reference coupons were not treated as laminate prediction targets. Instead, their full stress–strain curves were used later to construct the pointwise constituent-anchor mean curves and standard-deviation envelopes described in Section 2.4.

The complete coupon dataset was divided according to its role in the workflow, as summarized in Table 2. Monolithic PETG and PC-ABS coupons were used to define the constituent-material anchors. Laminate C1 and Laminate C2 were used to evaluate zero-shot prediction, laminate learning, and out-of-distribution (OOD) validation. The Rank 1–3 coupons were used in the final model-guided architecture validation stage.

The first PETG/PC-ABS laminate introduced into the learning workflow, denoted Laminate C1, was the 0.2 mm alternating-layer architecture starting with PETG on the build plate. This configuration was selected because 0.2 mm was the layer height used for the laminate slicing condition, and alternating the two materials at this layer scale produced the most interface-rich configuration within the 4 mm coupon thickness. As a result, the architecture contained the maximum number of PETG/PC-ABS material transitions available for the selected laminate build strategy. Introducing this case first allowed the algorithm to encounter an intentionally demanding interface-sensitive laminate response before being applied to lower-interface or model-suggested architectures. Therefore, Laminate C1 was used as the initial zero-shot comparison case and then retained in the sequential learning memory to provide the first laminate-specific residual correction beyond the constituent-material rule-of-mixtures baseline [7].

**Figure 2:**
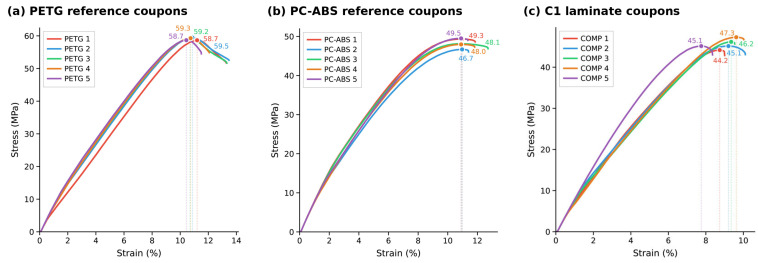
Experimental tensile stress–strain curves used for model inputs and initial laminate learning: (**a**) PETG single-material reference coupons, (**b**) PC-ABS single-material reference coupons, and (**c**) C1 PETG/PC-ABS laminate coupons.

The experimental tensile records used for the reference and initial laminate data acquisition are illustrated in Figure 2.

#### 2.2.2. Coupon Geometry

**Figure 3:**
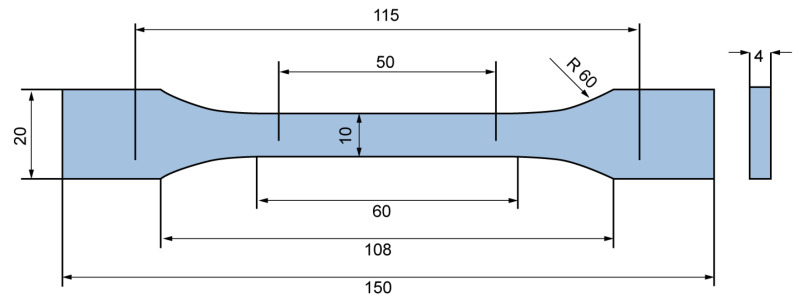
Dog-bone-shaped Type 1B standard coupons according to ISO 527-2. Dimensions are given in mm.

All coupons used in this research were prepared according to ISO 527-2, the dog-bone-shaped Type 1B tensile coupons [48]. The nominal coupon thickness was maintained at 4 mm for all coupon groups. The principal coupon dimensions, including the 150 mm overall length, 50 mm gauge length, 10 mm reduced-section width, 20 mm end width, R60 transition radius, and 4 mm thickness, are shown in Figure 3.

The coupon geometry shown in Figure 3 was adopted from the previously reported PETG/PC-ABS MEX laminate study and was retained in the present work as the common geometry for both single-material reference testing and subsequent laminate validation [7]. The use of a consistent coupon geometry is important here because the constituent-material anchor curves constructed later in Section 2.4 and the laminate curves used for residual learning are compared on the same tensile-coupon basis.

#### 2.2.3. MEX Fabrication Conditions

The material properties and fabrication settings used for the pure-material and PETG/PC-ABS laminate coupon groups are summarized in Table 3. The table is included here to define the material inputs and fabrication conditions used to generate the experimental coupon data; mechanical performance interpretation is not discussed in this section.

#### 2.2.4. Tensile Testing Protocol

The tensile-test data used in this study were taken from the repository associated with the previously published PETG/PC-ABS MEX laminate characterization study [49]. In that study, tensile testing was conducted according to ISO 527-2 using Type 1B dog-bone coupons printed from PETG, PC-ABS, and PETG/PC-ABS laminate configurations. The tests were performed on a Shimadzu EZ-LX universal testing machine equipped with a 5 kN load cell and operated under displacement control. All tensile tests used in the present work were conducted at a crosshead speed of 50 mm/min.

The dataset used for model construction and validation included five monolithic PETG coupons and five monolithic PC-ABS coupons for anchor-curve construction. The 0.2 mm alternating PETG/PC-ABS laminate contained five coupons and was used for the initial zero-shot comparison and interface-rich learning case. The 1.33 mm PC-ABS/PETG/PC-ABS laminate contained two coupons and was used as the primary out-of-distribution validation case. The three model-suggested ranked laminate architectures were subsequently printed and tested using three coupons per architecture.

The raw tensile records consisted of stress–strain data exported from the mechanical testing workflow. These full stress–strain curves, rather than only scalar mechanical properties, were used for anchor-curve construction, residual learning, and prediction validation. Strain was inferred from crosshead displacement in the original testing workflow, with no extensometer reported. Therefore, the present study treats the exported stress–strain records as machine-derived tensile response curves and applies the quality assurance and preprocessing steps described in Section 2.3 prior to model training or validation.

### 2.3. Data Acquisition, Quality Assurance, and Preprocessing

The raw tensile records were not used directly for model training or validation. Instead, each coupon file was passed through a standardized preprocessing workflow to obtain clean and comparable stress–strain curves across monolithic PETG, monolithic PC-ABS, and PETG/PC-ABS laminate coupons. The workflow identified the available force, stress, and strain channels, removed non-numeric metadata rows, corrected strain units where required, removed the preload region, and organized each curve into a consistent strain-increasing format. The resulting cleaned tensile curves were then used for anchor-curve construction, residual learning, phase detection, and full-curve prediction assessment.

#### 2.3.1. Raw Coupon Data Acquisition

For each tensile coupon, the exported mechanical test record was treated as the raw input to the computational workflow. The records contained tabulated tensile-response data, including stress and strain, with force also available in the relevant files. Only individual coupon-level records were used for curve-level analysis; summary files, statistical reports, and previously processed output files were excluded to avoid mixing raw experimental measurements with aggregated results. The full stress–strain curves were retained throughout preprocessing because the proposed framework learns and validates the complete tensile response rather than relying only on scalar outputs such as ultimate tensile strength.

#### 2.3.2. Column Detection and File Loading

Each coupon file was loaded using an automated column-identification step to account for possible differences in exported column names or ordering. Stress and strain channels were identified from the column headings using keyword matching, while derived quantities such as modulus were excluded from stress selection. When a force channel was present, it was also retained because it provided additional information for preload removal and quality checking. After the relevant channels were identified, the selected data were converted to numeric form, rows with missing or invalid values were removed, and files without usable stress–strain information were excluded from further analysis.

#### 2.3.3. Strain-Unit Conversion and Interpolation

After the stress and strain channels were extracted, the strain values were checked for unit consistency. Records reported in percentage strain were converted to decimal strain so that all coupons used the same strain representation. The stress–strain data were then sorted in increasing strain order, and duplicate strain entries were removed to produce a monotonic curve suitable for interpolation and comparison. The cleaned monolithic PETG and PC-ABS curves were interpolated onto a common strain grid for pointwise calculation of mean anchor curves and variability bands, while laminate coupon curves were later projected onto the model grid used for residual computation and validation.

#### 2.3.4. Coupon Quality Checks and Rejection Criteria

Quality assurance was applied before any coupon curve was used for anchor construction, learning, or validation. Files were rejected if they did not contain valid stress–strain data or if too few usable points remained after numerical cleaning. When force data were available, the initial preload region was removed using the early low-force portion of the curve, and the force–stress relationship was checked for consistency as an indicator of possible measurement instability. Coupons showing unrecoverable data-format problems were excluded, while coupons with usable stress–strain curves but minor warnings were retained after preprocessing. This ensured that the learning framework was trained and evaluated using physically meaningful tensile curves rather than corrupt or incomplete records.

### 2.4. Constituent-Material Anchors and Rule-of-Mixtures Baseline

The cleaned PETG and PC-ABS single-material reference curves were interpolated to a common strain grid. Pointwise mean curves were used as the constituent-material anchor curves, and pointwise standard-deviation curves were retained to describe reference-coupon variability. For a laminate stack with layer thicknesses $t_{l}$, the PETG fraction was calculated from the total PETG thickness divided by the total stack thickness:

(2) $$r=\frac{\sum_{l}I_{l}^{\mathrm{PETG}}t_{l}}{\sum_{l}t_{l}},\;I_{l}^{\mathrm{PETG}}=1\;\mathrm{for}\;\mathrm{PETG},\;0\;\mathrm{for}\;\mathrm{PC}-\mathrm{ABS}.$$

When mass ratio is required, it can be calculated from the same stack description by density weighting:

(3) $$r_{m}=\frac{\rho_{\mathrm{PETG}}\sum_{l}I_{l}^{\mathrm{PETG}}t_{l}}{\rho_{\mathrm{PETG}}\sum_{l}I_{l}^{\mathrm{PETG}}t_{l}+\rho_{\mathrm{PCABS}}\sum_{l}\left(1-I_{l}^{\mathrm{PETG}}\right)t_{l}},$$

where $\rho_{\mathrm{PETG}}=1.27$ g/cm^3^ and $\rho_{\mathrm{PCABS}}=1.07$ g/cm^3^ are the density values used in Table 3. The implemented residual-learning model used the thickness fraction *r* as its composition input because the candidate stacks were generated by layer thickness. The density-weighted mass ratio $r_{m}$ was used only for mass-related derived quantities and is a direct extension for future versions of the model. The initial laminate prediction was the material-fraction-weighted RoM curve:

(4) $$\sigma_{\mathrm{RoM}}\left(\varepsilon ;r\right)=r\sigma_{p}^{\mathrm{PETG}}\left(\varepsilon \right)+\left(1-r\right)\sigma_{p}^{\mathrm{PCABS}}\left(\varepsilon \right).$$

The reference variability curves were propagated using the same fraction weighting to provide a RoM uncertainty band for visualization. Before any laminate data had been learned, mixed PETG/PC-ABS stacks were additionally multiplied by a fixed adhesion-efficiency fallback of 0.83 for 0.1<r<0.9, while pure-material limits used a factor of 1.0. This value was not optimized during the validation sequence; it was an explicit zero-shot penalty introduced to avoid treating an interface-rich laminate as an ideal perfectly bonded mixture before any laminate evidence was available. The value is consistent with the tensile-strength reduction observed for alternating PETG/PC-ABS laminates in the preceding experimental characterization of this material pair [7]. Once laminate coupons had been accepted into the learning memory, predictions were generated by adding the learned residual correction to the unpenalized RoM baseline, and the 0.83 fallback was no longer used.

### 2.5. Mechanical Phase Detection and Adaptive Strain Grid

Each cleaned tensile curve was smoothed using a Savitzky–Golay filter implemented with scipy.signal.savgol_filter from SciPy version 1.17.0, with an adaptive odd window length up to 21 points and polynomial order 3. Force was used for gradient-based detection when available; otherwise, stress was used. The UTS location was identified as the maximum of the smoothed stress curve, and the elastic-end location was detected from the pre-UTS gradient as the first point after the peak slope where the gradient dropped below 50% of that peak. Points before the elastic end were labeled elastic, points between the elastic end and UTS were labeled hardening, and points at or after UTS were labeled post-UTS/necking. Because strain was derived from crosshead displacement rather than an extensometer or digital image correlation, these phase labels should be interpreted as algorithmic curve-region labels used for weighting and feature construction, not as direct local-strain measurements.

Characteristic curve anchors were extracted for later preprocessing and prediction support: elastic modulus, 0.2% offset yield point, UTS stress and strain, post-UTS necking rate, fracture stress and strain, toughness up to UTS, total toughness, elastic-end index, and UTS index. These anchors were not used as scalar prediction targets; they were used to describe the curve, guide the adaptive grid, and support OOD phase-label assignment.

The adaptive strain grid retained a uniform baseline resolution and added refinement near mechanically important regions, including yield, UTS, phase boundaries, and high-curvature zones. In the supplied implementation, the reference curves were first represented on a fixed 750-point standardized grid, while the residual model used an internal adaptive grid with a base resolution of 0.0005 strain, local refinement around curvature and phase-boundary zones, and a cap of 3000 points. Whenever a newly accepted coupon extended the strain range or introduced new refinement zones, the grid was updated and all stored coupons were reprojected onto the updated grid. Training points were also assigned phase-aware weights: elastic and post-UTS/necking regions were upweighted, and an additional weight was added near the UTS stress. The weight vector was normalized to keep the average sample weight near one. The present dataset supports the implemented adaptive-grid workflow, but it does not contain a separately reported fixed-grid ablation; therefore, the isolated quantitative contribution of grid adaptation is treated as a future ablation rather than inferred from the reported validation metrics.

### 2.6. Phase-Aware Residual Learning and Continual Updating

The correction model learned the pointwise difference between each measured laminate curve and the RoM baseline. For an accepted laminate coupon *c*, the experimental curve was interpolated onto the current adaptive grid and the raw residual was defined as:

(5) $$\Delta \sigma_{c}\left(\varepsilon \right)=\sigma_{\exp,c}\left(\varepsilon \right)-\sigma_{\mathrm{RoM}}\left(\varepsilon ;r_{c}\right).$$

Residuals were normalized before training using the laminate mixing factor 4r(1−r) and a local stress scale based on the RoM magnitude and coupon UTS. This normalization reduced the influence of near-zero stress regions and expressed the correction relative to the mixed-material condition. Each grid point was then represented as a supervised learning sample with four input features:

(6) $$x_{c}\left(\varepsilon \right)=\left[\varepsilon ,\;\sigma_{\mathrm{RoM}}\left(\varepsilon ;r_{c}\right),\;\phi_{c}\left(\varepsilon \right),\;r_{c}\right],$$

where $\phi_{c}\left(\varepsilon \right)$ is the local phase label. The model was implemented as a sample-weighted XGBoost regressor (Python xgboost package version 3.1.3) with objective reg:squarederror, n_estimators = 300, max_depth = 5, learning_rate = 0.04, subsample = 0.85, colsample_bytree = 0.85, n_jobs = 4, and random_state = 42. The phase-aware weights described above were supplied as sample weights during fitting. Although each grid point was represented as a supervised sample, the effective experimental replication remained the number of physical coupons, not the number of grid points. The pointwise representation was used to learn curve shape, while performance was reported at coupon level on sequential blind or pre-update predictions to reduce the risk of overstating the training size.

For OOD prediction after learning, the model predicted a normalized residual correction on the requested strain grid. The normalized output was converted back to a stress correction using the same mixing factor, added to the RoM baseline, and clipped to non-negative stress:

(7) $$\sigma_{\mathrm{pred}}\left(\varepsilon ;r\right)=\max\left[\sigma_{\mathrm{RoM}}\left(\varepsilon ;r\right)+\Delta \sigma_{\mathrm{learned}}\left(\varepsilon ;r\right),\;0\right].$$

Continual updating was performed by sequentially ingesting newly tested laminate coupons. Each accepted coupon was stored with its raw strain, stress, optional force, phase labels, anchors, PETG fraction, and source identifier. The model was not updated using only the newest coupon; instead, after each accepted coupon, the complete stored coupon memory was reprojected onto the current adaptive grid and the residual model was refitted from all accepted evidence. Coupons were rejected before memory insertion if they had very low UTS, UTS at the curve end, excessive negative stress values, non-zero initial strain beyond the accepted tolerance, or non-positive elastic modulus.

### 2.7. Evolving Continual-Learning Algorithm

The implemented framework was an evolving digital-twin algorithm rather than a fixed offline predictor. In the zero-shot state, the laminate response was estimated only from the constituent-material anchors and the RoM baseline. After a new laminate coupon was tested, its full stress–strain curve was cleaned, phase-labeled, checked for physical plausibility, and added to the continual-learning memory. The update therefore used the complete curve shape rather than only a scalar property such as UTS.

For every accepted coupon, the algorithm calculated the pointwise residual between the measured laminate curve and the RoM prediction. Each point on the adaptive grid was represented by strain, RoM stress, mechanical phase label, and PETG fraction. The phase label allowed the correction model to learn different residual patterns in the elastic, hardening, and post-UTS/necking regions.

The strain grid also evolved during learning. If a newly accepted coupon extended the observed strain range or introduced new high-curvature, yield, or UTS regions, the grid was rebuilt with additional points near these mechanically important zones. Previously accepted coupons were not discarded. Instead, their raw curves were reprojected onto the updated grid, and the residual model was refitted using all accumulated coupon evidence. Thus, each new coupon updated both the training memory and, when necessary, the numerical grid on which the full-curve correction was learned.

The evolving update sequence was:

1. construct PETG and PC-ABS constituent-anchor curves from monolithic coupons;
2. generate the initial RoM prediction for the selected laminate stack;
3. load a new laminate coupon and apply preprocessing, phase detection, and plausibility checks;
4. update the adaptive strain grid when the coupon adds a wider strain range or new mechanically important zones;
5. store the accepted raw coupon in the continual-learning memory;
6. reproject all stored coupons onto the current adaptive grid;
7. refit the phase-aware residual model using pointwise samples and sample weights;
8. add the learned residual correction to the RoM baseline for subsequent OOD predictions.

After each accepted coupon, the workflow produced a before–after prediction comparison using full-curve RMSE and UTS agreement. This made it possible to evaluate whether the digital twin improved not only the peak-stress prediction but also the complete stress–strain trajectory. The framework therefore progressed from a constituent-material-based estimate toward an architecture-informed laminate predictor as experimental evidence accumulated.

### 2.8. OOD Laminate Screening and Ranking

OOD candidates were represented as ordered material-thickness stacks. For automated screening, 20 layers of 0.2 mm were used to give the same 4 mm nominal coupon thickness as the experiments. Mixed PETG/PC-ABS stacks were generated by varying the PETG layer count from 1 to 19 and randomly shuffling the layer order with seed 42. For each PETG count, up to 526 unique stacks were retained, with a maximum of 2000 shuffle attempts. In the current residual model, stack order was used to define candidate architectures and experimental fabrication, but the learned pointwise features used PETG thickness fraction rather than explicit sequence descriptors. Therefore, stacks with identical PETG fractions but different order are not fully distinguishable by the present regressor until architecture-specific coupon data are added.

For every candidate stack, the PETG fraction was calculated, the RoM curve was generated, and the final stress–strain curve was predicted either by the zero-shot adhesion-corrected RoM branch or by the learned residual-corrected branch. The OOD engine also returned a two-sigma prediction envelope by combining Gaussian-process-derived constituent sigma curves using the same PETG/PC-ABS fractions. This band was used only as a prediction-support envelope and was not treated as a measured experimental confidence interval.

Derived screening quantities were calculated directly from the predicted stress–strain curve. Predicted UTS was taken as the maximum predicted stress, elongation was taken at the predicted UTS point, stroke was calculated using a 50 mm gauge length, force was calculated from stress multiplied by the cross-sectional area (10 mm width multiplied by stack thickness), and coupon weight was estimated from the fraction-weighted full-coupon weights of PETG (10.3 g) and PC-ABS (8.5 g). Candidate architectures were ranked using the implemented UTS–elongation score, selected as a simple strength–ductility proxy for pre-fabrication screening:

(8) $$S_{q}=\sigma_{\mathrm{UTS},\mathrm{pred},q}\;\varepsilon_{\mathrm{UTS},\mathrm{pred},q}^{\left(\%\right)}.$$

The final Rank 1–Rank 3 designs were the highest-scoring predicted candidates after enforcing uniqueness of the rounded PETG fraction. Toughness and multi-objective ranking were not used as the primary selection rule because the reported error metric emphasized the curve up to UTS and because post-UTS behavior is more sensitive to localization when strain is derived from crosshead displacement. These ranked designs were then fabricated and tested for model-guided validation.

### 2.9. Experimental Validation and Error Metrics

Validation was performed in three stages. First, Laminate C1 was predicted in zero-shot mode using only constituent anchors and was then introduced into the learning memory coupon by coupon. Second, Laminate C2 was used as the primary OOD validation case after laminate learning. Third, the model-guided Rank 1–Rank 3 architectures were printed and tested to evaluate the screening workflow on newly suggested stacks.

For each tested coupon, the prediction generated before learning that coupon was retained as the blind or zero-shot prediction. After testing and quality acceptance, the coupon was added to memory, the model was refitted, and the updated prediction was generated. The protocol can be summarized as: predict → test → compare → accept → update memory → refit model.

UTS error was calculated from the difference between predicted and experimental peak stress. Full-curve accuracy was evaluated on a common strain basis after interpolation. The comparison region was restricted to experimental stress values of at least 1 MPa and strains up to the experimental UTS strain. Pointwise relative stress errors $e_{i}$ were then used to calculate RMSE and MAE percentages:

(9) $$\begin{array}{cc} \mathrm{UTS}\;\mathrm{error}(\%) & =100\frac{\sigma_{\mathrm{UTS},\mathrm{pred}}-\sigma_{\mathrm{UTS},\exp}}{\sigma_{\mathrm{UTS},\exp}},\\ \mathrm{RMSE}(\%) & =100\sqrt{\mathrm{mean}(e_{i}^{2})},\\ \mathrm{MAE}(\%) & =100\mathrm{mean}(|e_{i}|). \end{array}$$

These metrics were selected because UTS error measures the peak-stress prediction, whereas RMSE and MAE evaluate agreement across the tensile trajectory up to UTS. The upper limit at experimental UTS strain was selected because pre-UTS behavior is the most comparable load-bearing part of the curve for these displacement-controlled tests; after UTS, necking, crack propagation, and failure localization can cause larger crosshead-displacement artifacts and coupon-specific fracture-path effects. This distinction is important for PETG/PC-ABS laminates because similar peak stress can occur with different curve shapes, stiffness evolution, and load-transfer behavior.

## 3. Results

This section presents the validation results of the proposed adaptive continual-learning framework for full tensile-curve prediction of PETG/PC-ABS laminate architectures. The validation was conducted in three stages using the architectures defined in Section 2: Laminate C1, Laminate C2, and the three model-suggested architectures denoted Rank 1, Rank 2, and Rank 3. Laminate C1 was used for the initial zero-shot comparison and as the interface-rich learning case. Laminate C2 was used as the primary out-of-distribution validation architecture after laminate learning. Finally, Rank 1–3 were generated through the model-based screening step, fabricated, and tested experimentally to evaluate the predictive capability of the workflow on suggested laminate designs.

Prediction accuracy was evaluated using both scalar and full-curve metrics. UTS error was used to quantify peak-stress prediction accuracy, whereas full-curve RMSE and MAE were used to assess agreement between the predicted and experimental stress–strain trajectories. Full-curve errors were calculated from the stress threshold of 1 MPa up to the experimentally observed UTS strain for each coupon. This evaluation range excludes near-zero stress instabilities while preserving the loading region relevant to stiffness evolution, nonlinear transition, and peak-stress development.

The results are presented according to the three validation stages: zero-shot prediction using Laminate C1, OOD validation using Laminate C2, and experimental validation of the model-suggested Rank 1–3 architectures. This organization follows the workflow described in Section 2 and separates the baseline predictive capability, learned OOD transfer, and model-guided design validation stages.

### 3.1. Zero-Shot Prediction and Initial Learning Using Laminate C1

**Figure 4:**
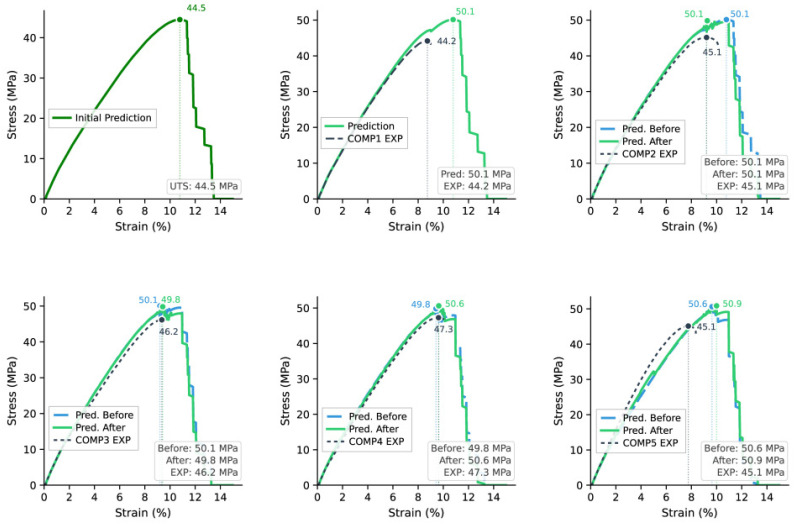
Sequential full-curve prediction response for Laminate C1. The zero-shot prediction was generated using only constituent-material anchors, followed by updated predictions after each coupon was introduced into the learning sequence.

Laminate C1 was first used to evaluate the zero-shot capability of the framework before any laminate coupon data were introduced into the learning system. At this stage, the prediction was generated only from the constituent-material anchors and the rule-of-mixtures-based baseline described in Section 2. Figure 4 shows the initial Laminate C1 prediction and the subsequent prediction updates obtained as each coupon was incorporated into the learning sequence.

The corresponding quantitative accuracy metrics for Laminate C1 are summarized in Table 4, where the first prediction was generated without laminate coupon data, while subsequent predictions were updated after each Laminate C1 coupon was introduced into the learning sequence.

For the zero-shot prediction, the model predicted a UTS of 44.49 MPa for COMP1, compared with the experimental value of 44.16 MPa. This corresponds to a UTS error of only +0.74%. However, the full-curve RMSE and MAE were 12.34% and 12.24%, respectively, indicating that the close agreement in peak stress did not correspond to equally accurate prediction of the complete stress–strain trajectory. This result shows that the constituent-based baseline provided a reasonable first estimate of the peak response but still retained a measurable full-curve mismatch before any laminate data were learned.

After COMP1 was introduced into the model, the updated prediction showed a substantial improvement in full-curve accuracy. The RMSE decreased from 12.34% to 2.72%, while the MAE decreased from 12.24% to 2.25%. This was the largest single reduction in error observed within the Laminate C1 sequence. The predicted UTS after learning COMP1 increased to 50.13 MPa, producing a UTS error of +13.52%. Therefore, although the full-curve agreement improved markedly after the first learning step, the peak-stress prediction became more conservative relative to the experimental UTS.

The subsequent blind predictions for COMP2–COMP4 showed that the correction learned from the earlier coupons transferred to unseen coupons within the same Laminate C1 architecture. Before learning COMP2, the model produced a full-curve RMSE of 3.42% and MAE of 2.96%. After COMP2 was incorporated, the corresponding self-validation errors decreased to 2.86% and 2.52%, respectively.

For COMP3, the blind and updated predictions gave nearly identical RMSE values of 4.49%, with MAE values of 4.13% and 4.14%.

For COMP4, the blind prediction gave an RMSE of 5.51% and MAE of 4.69%, which decreased to 4.63% and 3.99% after COMP4 was incorporated. These results indicate that, after the first coupon, the model maintained relatively stable full-curve prediction accuracy for COMP2–COMP4.

COMP5 showed a different response from the preceding coupons. The blind prediction before learning COMP5 produced a full-curve RMSE of 11.66% and MAE of 10.94%, which were substantially higher than the corresponding errors for COMP2–COMP4. After COMP5 was incorporated, the updated prediction reduced the RMSE to 9.33% and the MAE to 8.87%; however, these values remained higher than the updated errors obtained for the previous coupons. The predicted UTS for COMP5 was 50.87 MPa compared with the experimental UTS of 45.13 MPa, corresponding to a final UTS error of +12.71%.

Overall, the Laminate C1 results show that the zero-shot baseline provided a close initial UTS estimate but a larger full-curve error. Incorporating the first laminate coupon produced the dominant improvement in stress–strain curve prediction, reducing the RMSE by approximately 78%. Subsequent updates produced smaller changes for COMP2–COMP4, whereas COMP5 retained a larger full-curve error even after learning. As shown in Figure 4 and Table 4, this first validation stage demonstrates the transition from a constituent-based zero-shot prediction to a coupon-informed prediction model for an interface-rich laminate architecture.

### 3.2. OOD Validation After Composite Learning Using Laminate C2

**Figure 5:**
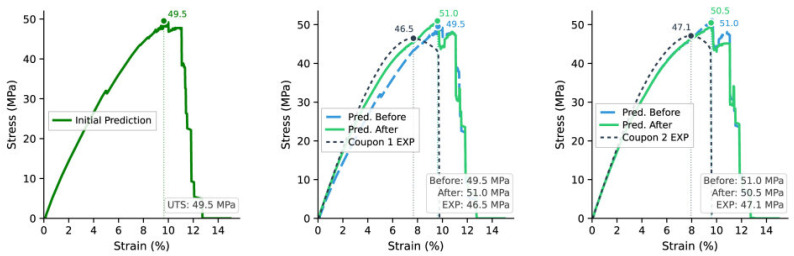
Sequential predicted and experimental stress–strain responses for Laminate C2 during blind OOD prediction and subsequent coupon-based updates.

Laminate C2 was used to evaluate the out-of-distribution prediction capability of the framework after the model had been exposed to laminate learning data, but before any Laminate C2 experimental coupon was introduced. Therefore, the initial Laminate C2 prediction represents a blind OOD case for this architecture. Figure 5 compares the predicted and experimental stress–strain responses for the Laminate C2 coupon sequence.

Table 5 summarizes the corresponding UTS error, full-curve RMSE, and full-curve MAE values.

For the blind OOD prediction against Exp1, the model predicted a UTS of 49.53 MPa, compared with the experimental UTS of 46.48 MPa. This corresponded to a UTS error of +6.56%. The full-curve RMSE and MAE were 18.60% and 18.22%, respectively, indicating that the initial Laminate C2 prediction retained a substantial mismatch over the pre-peak stress–strain trajectory. Thus, although the predicted peak stress was within approximately 7% of the experimental UTS, the complete curve comparison showed that the blind prediction did not yet reproduce the full tensile response accurately.

After Exp1 was incorporated into the learning sequence, the updated prediction showed a clear improvement in full-curve accuracy. The RMSE decreased from 18.60% to 6.38%, while the MAE decreased from 18.22% to 5.80%. This corresponds to an approximately 66% reduction in RMSE after learning a single Laminate C2 coupon. The predicted UTS after learning Exp1 was 50.96 MPa, giving a UTS error of +9.64%. Similar to the Laminate C1 case, the improvement in full-curve agreement was not accompanied by a reduction in UTS error, showing that peak-stress error and curve-shape error did not vary in the same manner.

The generalization capability of the learned model was then evaluated by applying the prediction obtained after Exp1 blindly to Exp2. For this unseen Laminate C2 coupon, the model achieved a full-curve RMSE of 6.22% and MAE of 5.69%, which were nearly identical to the self-validation errors obtained for Exp1. The predicted UTS was 50.96 MPa compared with the experimental UTS of 47.12 MPa, corresponding to a UTS error of +8.15%. The close agreement between the Exp1 self-validation error and the blind Exp2 error indicates that the correction learned from the first Laminate C2 coupon transferred to the second coupon without a substantial loss in full-curve accuracy.

After Exp2 was incorporated, the final updated prediction gave a full-curve RMSE of 6.19% and MAE of 5.67%. These values were only slightly lower than the blind prediction errors for Exp2, showing that most of the achievable full-curve improvement had already occurred after learning Exp1. The predicted UTS after learning Exp2 decreased to 50.48 MPa, reducing the UTS error from +8.15% to +7.13%.

Overall, the Laminate C2 validation shows that the initial blind OOD prediction had a relatively high full-curve error, but a single architecture-specific coupon reduced the RMSE from 18.60% to approximately 6.4%. The subsequent blind prediction on Exp2 maintained essentially the same full-curve accuracy, and the final update after Exp2 produced only a marginal additional reduction. As shown in Figure 5 and Table 5, Laminate C2 therefore demonstrates rapid adaptation after the first coupon and stable generalization to a second unseen coupon within the same architecture.

### 3.3. Model-Guided Validation of Ranked Architectures

After Laminate C1 and Laminate C2 validation, the trained framework was used to screen candidate laminate configurations and suggest the top-ranked architectures for experimental testing. The selected architectures, denoted Rank 1, Rank 2, and Rank 3, were fabricated and tested as described in Section 2. This stage evaluates whether the model-guided screening workflow could generate experimentally testable laminate designs and maintain predictive accuracy when applied to newly suggested architectures.

**Figure 6:**
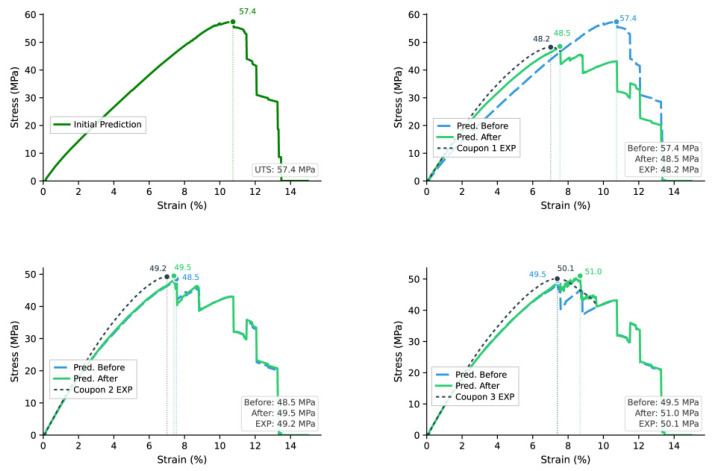
Sequential predicted and experimental stress–strain responses for Rank 1 during blind prediction and subsequent coupon-based updates.

For Rank 1, the initial blind prediction showed the largest baseline mismatch among the three ranked architectures. The predicted and experimental stress–strain responses of Rank 1 are shown in Figure 6, and the corresponding accuracy metrics are summarized in Table 6.

The predicted UTS was 57.40 MPa compared with the experimental UTS of 48.21 MPa for Coupon 1, giving a UTS error of +19.06%. The corresponding full-curve RMSE and MAE were 21.04% and 20.67%, respectively. After Coupon 1 was introduced into the learning sequence, the full-curve RMSE decreased to 6.93% and the MAE decreased to 6.47%. The predicted UTS also shifted to 48.50 MPa, reducing the UTS error to +0.60%. Subsequent predictions for Coupon 2 and Coupon 3 remained within a narrower full-curve error range. The blind prediction for Coupon 2 gave an RMSE of 8.30%, which decreased to 7.59% after Coupon 2 was learned. For Coupon 3, the blind prediction gave an RMSE of 6.51%, while the updated prediction after learning Coupon 3 gave an RMSE of 6.66%. Thus, Rank 1 showed strong first-coupon correction followed by relatively stable full-curve prediction accuracy across the remaining coupons.

**Figure 7:**
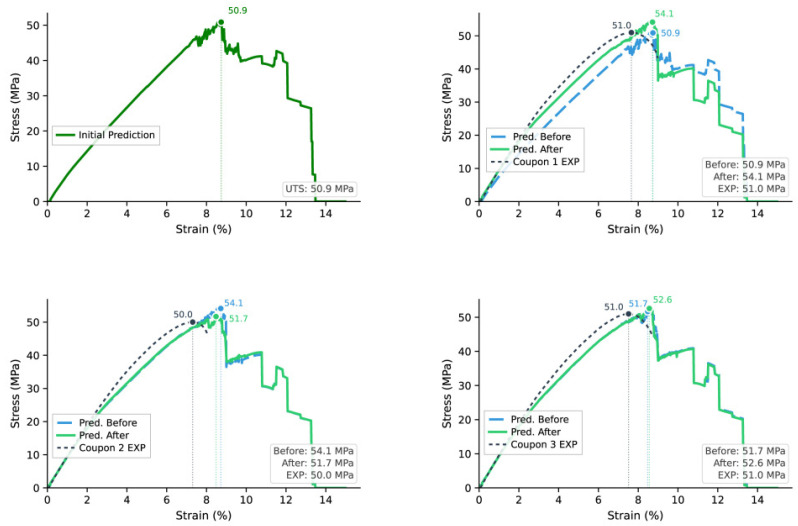
Sequential predicted and experimental stress–strain responses for Rank 2 during blind prediction and subsequent coupon-based updates.

For Rank 2, the blind prediction showed a different behavior. The predicted and experimental stress–strain responses of Rank 2 are shown in Figure 7, and the corresponding accuracy metrics are summarized in Table 7.

The initial predicted UTS was 50.88 MPa, which was very close to the experimental UTS of 50.98 MPa for Coupon 1, corresponding to a UTS error of −0.18%. However, the full-curve RMSE and MAE were still high, at 20.74% and 20.25%, respectively. After Coupon 1 was introduced, the RMSE decreased to 7.50% and the MAE decreased to 6.67%, while the predicted UTS increased to 54.13 MPa. For Coupon 2, the blind prediction gave an RMSE of 8.34%, and the updated prediction after learning Coupon 2 gave an RMSE of 8.21%. For Coupon 3, the blind prediction gave an RMSE of 8.06%, which decreased to 7.71% after Coupon 3 was incorporated. Across Rank 2, the full-curve RMSE remained within approximately 7.5–8.3% after the first coupon update, indicating that most of the improvement occurred during the first learning step.

**Figure 8:**
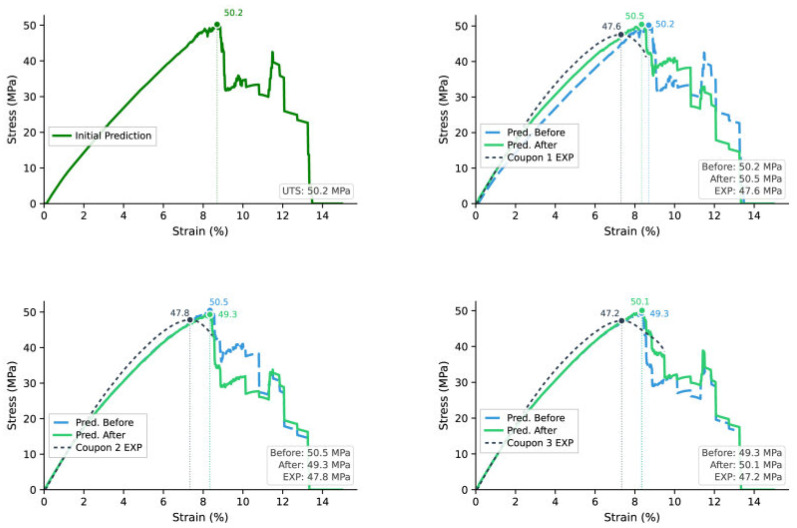
Sequential predicted and experimental stress–strain responses for Rank 3 during blind prediction and subsequent coupon-based updates.

For Rank 3, the initial blind prediction produced a predicted UTS of 50.23 MPa compared with the experimental UTS of 47.58 MPa for Coupon 1, corresponding to a UTS error of +5.58% as shown in Figure 8.

The corresponding full-curve RMSE and MAE were 19.40% and 18.65%, respectively. After Coupon 1 was introduced, the RMSE decreased to 7.48% and the MAE decreased to 6.43% (see Table 8).

**Figure 9:**
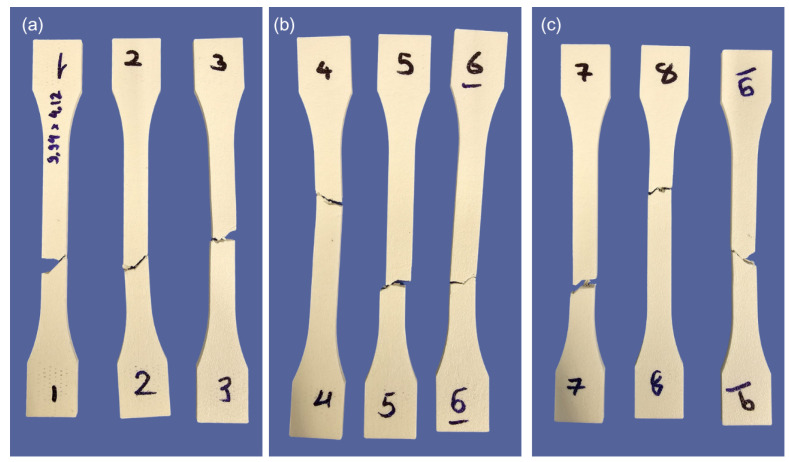
Experimentally tested fractured tensile coupons for the model-suggested laminate architectures: (**a**) Rank 1, (**b**) Rank 2, and (**c**) Rank 3. Each panel shows the three repeated coupons tested for the corresponding ranked architecture after tensile loading.

The experimentally tested fractured coupons corresponding to the three model-suggested ranked architectures are shown in Figure 9.

The blind prediction for Coupon 2 gave nearly the same full-curve accuracy, with RMSE and MAE values of 7.46% and 6.45%, respectively. After learning Coupon 2, the RMSE was 7.74%. For Coupon 3, the blind prediction gave the lowest RMSE in the Rank 3 sequence, at 7.24%, while the updated prediction after Coupon 3 gave an RMSE of 7.53%. Overall, Rank 3 showed rapid convergence after the first coupon, with subsequent full-curve RMSE values remaining within a narrow range of 7.24–7.74%.

Taken together, the ranked-architecture validation results show that all three model-suggested architectures experienced a large reduction in full-curve error after the first coupon was introduced. The initial blind RMSE values were 21.04%, 20.74%, and 19.40% for Rank 1, Rank 2, and Rank 3, respectively. After the first coupon update, these values decreased to 6.93%, 7.50%, and 7.48%. Additional coupon updates produced smaller changes, with the post-learning RMSE values generally remaining between approximately 6.5% and 8.3%. This ranked-architecture stage therefore shows that the model-guided screening process generated printable and experimentally testable designs, and that the framework could adapt rapidly to each selected architecture after a single coupon observation.

## 4. Discussion

The results show a consistent transition from constituent-based zero-shot or blind OOD predictions to coupon-informed full-curve predictions. The largest reduction in full-curve RMSE generally occurred after the first architecture-specific coupon was introduced. This does not prove broad statistical robustness, because the available coupon population was limited, but it does demonstrate a data-efficient sequential-learning behavior for the tested PETG/PC-ABS laminate system.

### 4.1. Predictive Scope and OOD Interpretation

The three-stage validation sequence separates three capabilities. Laminate C1 tested the zero-shot response generated from constituent-material anchors. Laminate C2 tested transfer to an unseen laminate after learning had started on a different architecture. Rank 1–3 were selected before fabrication by the screening workflow and therefore represent a prospective design-selection step at the time of their first prediction. After coupon data from any ranked architecture were added, the later results should be interpreted as architecture-specific adaptation rather than purely prospective prediction.

This distinction is important for avoiding overstatement. The present model generalizes most directly across PETG thickness fraction and stress–strain phase behavior; it adapts after architecture-specific evidence is observed. Because explicit layer-sequence descriptors were not included in the XGBoost feature vector, two stacks with identical PETG fractions but different layer ordering are not fully distinguishable before coupon feedback. Future versions should add sequence descriptors such as the number of PETG/PC-ABS interfaces, first and last material, interface locations through the thickness, and density-weighted composition.

### 4.2. Full-Curve Error Versus UTS Error

UTS accuracy and full-curve accuracy were not always coupled. In the zero-shot prediction for Laminate C1, the UTS error was only +0.74%, while the full-curve RMSE was 12.34%. Rank 2 showed an even clearer example: the zero-shot UTS error was −0.18%, but the full-curve RMSE was 20.74%. Conversely, after learning, full-curve RMSE sometimes decreased while UTS error increased. This behavior is expected because the model objective minimizes weighted pointwise residuals across the curve rather than directly optimizing the single maximum-stress value. A prediction can therefore improve the global trajectory while shifting the peak slightly upward or downward.

### 4.3. Sequential Learning and Comparison with Baselines

The implemented comparison to a simpler baseline is the transition from the constituent-material RoM/zero-shot prediction to the learned residual-corrected prediction. The first coupon update reduced RMSE from 12.34% to 2.72% for Laminate C1, from 18.60% to 6.38% for Laminate C2, from 21.04% to 6.93% for Rank 1, from 20.74% to 7.50% for Rank 2, and from 19.40% to 7.48% for Rank 3. These values show the added value of laminate-specific residual learning relative to the blind baseline. However, the supplied results do not include a full fixed-grid, no-phase, or no-weight ablation. The study therefore reports only the baseline comparison supported by the available data and identifies formal ablation as future work.

### 4.4. Interface Interpretation

The curve shapes and the deviations from ideal RoM behavior are consistent with interface-sensitive load transfer in PETG/PC-ABS laminates, and this interpretation is supported by prior mechanical characterization of the same material pair [7]. Nevertheless, the present study did not perform microscopy, digital image correlation, or fracture-surface analysis. Therefore, local statements about voids, delamination, or fracture initiation should be treated as plausible explanations rather than directly observed mechanisms.

### 4.5. Extension to More Laminate Types and Other Loading Modes

Increasing the number of laminate types or extending the framework to more than two constituent materials is conceptually possible but requires additional descriptors and data. For additional materials, the feature vector would need a composition vector rather than a single PETG fraction, constituent-specific anchor curves for each material, and sequence descriptors that encode interface count and order. The required number of coupons would also increase because the model would need to distinguish material identity, composition, and architecture effects rather than only PETG/PC-ABS fraction-dependent behavior.

The current framework predicts tensile stress–strain curves only. Impact strength, compressive response, bending behavior, or fatigue strength should not be inferred from the present tensile model. These loading modes require their own experimental datasets, target definitions, phase or event-detection logic, and validation metrics. The same constituent-anchor and residual-learning concept could be transferred, but the present results do not validate those properties.

### 4.6. Limitations and Future Work

The main limitations are the small number of physical coupons, the use of crosshead-derived strain, the absence of explicit layer-sequence features in the residual regressor, the absence of microscopy or fracture-surface evidence, and the lack of formal fixed-grid/no-phase ablation. Future work should expand the coupon population, include extensometer or digital-image-correlation strain measurement, add sequence-aware descriptors, quantify fixed-grid and phase-label ablations, and validate the framework under impact, compression, bending, and fatigue loading.

## 5. Conclusions

This study developed and validated a constituent-material-anchored continual-learning framework for predicting the full tensile stress–strain response of PETG/PC-ABS laminate coupons fabricated by MEX. The framework begins from experimentally measured PETG and PC-ABS reference curves, constructs a RoM baseline, and then improves through phase-aware residual learning as laminate coupon data become available. The main conclusions are as follows:

- **A constituent-anchor prediction strategy was established.** PETG and PC-ABS tensile curves were used as physically interpretable reference anchors for the initial RoM baseline before architecture-specific laminate data were introduced.
- **Full-curve validation provided information that UTS alone could not capture.** Laminate C1 had a zero-shot UTS error of only +0.74%, but the full-curve RMSE was 12.34%. Rank 2 had a zero-shot UTS error of −0.18%, while the full-curve RMSE was 20.74%. Peak-stress agreement alone is therefore insufficient for evaluating tensile-curve prediction in multi-material laminates.
- **The first architecture-specific coupon produced the largest improvement.** RMSE decreased from 12.34% to 2.72% for Laminate C1, from 18.60% to 6.38% for Laminate C2, from 21.04% to 6.93% for Rank 1, from 20.74% to 7.50% for Rank 2, and from 19.40% to 7.48% for Rank 3 after the first coupon update.
- **Model-guided laminate screening was demonstrated as a prospective step.** Rank 1–3 were selected by the screening workflow before fabrication, printed, and tested. The subsequent coupon updates showed rapid architecture-specific adaptation, with post-first-update RMSE values generally near 7–8% for the ranked architectures.
- **The present scope is tensile and data-efficient, not universally robust.** The results support an interpretable proof of concept for PETG/PC-ABS tensile-curve prediction. Extension to more materials, identical-fraction sequence discrimination, impact or compressive properties, and statistically robust generalization requires additional descriptors and larger experimental datasets.

Overall, combining constituent-material anchors, a RoM baseline, phase-aware residual learning, and sequential coupon updates provides a practical route for full-curve tensile prediction and laminate screening in PETG/PC-ABS MEX. The framework is strongest as an iterative experimental-planning tool that improves after limited feedback while preserving transparent links to measured constituent-material behavior. 

## Figures and Tables

**Table 1:** Positioning of the present work relative to representative machine-learning-based mechanical-property prediction studies.

Study Class or Representative Studies	Typical Prediction Target	Multi-Material/ Interface Focus	Full Curve	OOD Architecture Test	Sequential Update
Reviews of ML in AM and polymer AM [1,2,3]	Process–property relationships, opportunities, and limitations	Partial	No	No	No
Early tensile-strength and parameter-optimization models [8,9,10,11,12,13,14]	UTS or optimized process settings	Partial	No	Mostly no	No
Ensemble, GP, boosting, and sensor-assisted polymer-AM models [17,22,23,24,25,26,27,28]	UTS, modulus, strain-at-break, surface or process-related properties	Partial	No or limited	Mostly no	Limited
Recent PETG, reinforced-polymer, and multi-material FFF studies [29,33,34,35,36,37,38,39,40,41,42]	Mechanical-property or tensile-strength prediction and optimization	Partial	No	Partial	No
Broader mechanical-capacity ML studies requested by the reviewer [43,44,45,46,47]	Lattice performance, casting properties, axial load capacity, compressive strength, or fatigue strength	No or system-specific	No or task-specific	System-specific	No
Present work	Full tensile stress–strain curve of PETG/PC-ABS MEX laminates	Yes	Yes	Yes	Yes

**Table 2:** Coupon datasets used for constituent-anchor construction, learning, OOD validation, and ranked-architecture testing.

Dataset Label	Coupon Group/Architecture	No. of Coupons	Role in Workflow	Main Use in the Present Study
PETG reference	Monolithic PETG coupons	5	Single-material reference input	Used to construct the PETG reference anchor curve, PETG variability curve, and PETG characteristic tensile response.
PC-ABS reference	Monolithic PC-ABS coupons	5	Single-material reference input	Used to construct the PC-ABS reference anchor curve, PC-ABS variability curve, and PC-ABS characteristic tensile response.
C1 laminate	0.2 mm alternating PETG/PC-ABS layers, starting from PETG on the bed	5	Interface-rich laminate dataset	Used for the initial zero-shot prediction/comparison and sequential learning from laminate coupon data.
C2 laminate	1.33 mm PC-ABS/1.33 mm PETG/1.33 mm PC-ABS	2	Primary OOD validation architecture	Used to evaluate prediction transfer after laminate learning on an unseen laminate architecture.
Rank 1	1.40 mm PETG/0.20 mm PC-ABS/2.40 mm PETG	3	Model-suggested architecture	Printed and tested to validate the highest-ranked architecture suggested by the screening workflow.
Rank 2	1.80 mm PETG/0.20 mm PC-ABS/0.40 mm PETG/0.20 mm PC-ABS/1.40 mm PETG	3	Model-suggested architecture	Printed and tested to validate the second-ranked architecture suggested by the screening workflow.
Rank 3	0.20 mm PETG/0.40 mm PC-ABS/0.40 mm PETG/0.20 mm PC-ABS/2.80 mm PETG	3	Model-suggested architecture	Printed and tested to validate the third-ranked architecture suggested by the screening workflow.

**Table 3:** Material properties and MEX fabrication settings used for PETG, PC-ABS, and PETG/PC-ABS laminate coupon groups. Adapted from the previous PETG/PC-ABS MEX laminate study [7].

Parameter	PETG Reference	PC-ABS Reference	Laminate C1	Laminate C2	Rank 1–3
Glass transition temperature	80 °C	115 °C	PETG/PC-ABS constituent values	PETG/PC-ABS constituent values	PETG/PC-ABS constituent values
Density	1.27 g/cm^3^	1.07 g/cm^3^	Layer-fraction dependent	Layer-fraction dependent	Layer-fraction dependent
Manufacturer-reported tensile strength	51 MPa	42 MPa	–	–	–
Nominal filament diameter	1.75 mm	1.75 mm	1.75 mm for both materials	1.75 mm for both materials	1.75 mm for both materials
Nozzle temperature	220–250 °C	260–270 °C	PETG layers: 220–250 °C; PC-ABS layers: 260–270 °C; nozzle held at 270 °C during filament changes	PETG layers: 220–250 °C; PC-ABS layers: 260–270 °C; nozzle held at 270 °C during filament changes	PETG layers: 220–250 °C; PC-ABS layers: 260–270 °C; nozzle held at 270 °C during filament changes
Bed temperature	90 °C	100 °C	70 °C	70 °C	70 °C
Print speed	50 mm/s	50 mm/s	40 mm/s	40 mm/s	40 mm/s
Cooling fan	Off	25% on	25% on for PC-ABS; off for PETG	25% on for PC-ABS; off for PETG	25% on for PC-ABS; off for PETG
Purge amount	∼1.2 g	∼1.2 g	∼5.5 g during material transition	∼5.5 g during material transition	∼5.5 g during material transition
Infill density	100%	100%	100%	100%	100%
Mechanical interlocking	Not used	Not used	Not used	Not used	Not used
Infill pattern	Rectilinear	Rectilinear	Rectilinear	Rectilinear	Rectilinear
Wall loops	2	2	2	2	2
Top and bottom shell layers	3	3	3	3	3
Nominal coupon thickness	4 mm	4 mm	4 mm	4 mm	4 mm

**Table 4:** Prediction accuracy of the sequential learning framework for Laminate C1.

Prediction Stage	Role in Learning Sequence	Ref. Exp.	Exp. UTS (MPa)	Pred. UTS (MPa)	UTS Error (%)	Full-Curve RMSE (%)	Full-Curve MAE (%)
**—Baseline (no learning)—**							
Predict 0	Blind OOD—no experimental data	COMP 1	44.16	44.49	+0.74%	12.34%	12.24%
**—After learning COMP1—**							
Predict 1	Self-validation after learning COMP1	COMP 1	44.16	50.13	+13.52%	2.72%	2.25%
Predict 1 (blind)	Blind prediction—before learning COMP2	COMP 2	45.14	50.13	+11.07%	3.42%	2.96%
**—After learning COMP2—**							
Predict 2	Updated prediction after learning COMP2	COMP 2	45.14	50.07	+10.93%	2.86%	2.52%
Predict 2 (blind)	Blind prediction—before learning COMP3	COMP 3	46.16	50.07	+8.47%	4.49%	4.13%
**—After learning COMP3—**							
Predict 3	Updated prediction after learning COMP3	COMP 3	46.16	49.82	+7.93%	4.49%	4.14%
Predict 3 (blind)	Blind prediction—before learning COMP4	COMP 4	47.28	49.82	+5.36%	5.51%	4.69%
**—After learning COMP4—**							
Predict 4	Updated prediction after learning COMP4	COMP 4	47.28	50.62	+7.07%	4.63%	3.99%
Predict 4 (blind)	Blind prediction—before learning COMP5	COMP 5	45.13	50.62	+12.17%	11.66%	10.94%
**—After learning COMP5—**							
Predict 5	Updated prediction after learning COMP5	COMP 5	45.13	50.87	+12.71%	9.33%	8.87%

**Table 5:** Sequential prediction accuracy for Laminate C2 before and after coupon-based learning.

Prediction Stage	Role in Learning Sequence	Ref. Exp.	Exp. UTS (MPa)	Pred. UTS (MPa)	UTS Error (%)	Full-Curve RMSE (%)	Full-Curve MAE (%)
**—Baseline (no learning)—**							
Predict 0	Blind OOD—no experimental data	Exp 1	46.48	49.53	+6.56%	18.60%	18.22%
**—After learning Exp1—**							
Predict 1	Self-validation after learning Exp1	Exp 1	46.48	50.96	+9.64%	6.38%	5.80%
Predict 1 (blind)	Blind prediction—before learning Exp2	Exp 2	47.12	50.96	+8.15%	6.22%	5.69%
**—After learning Exp2—**							
Predict 2	Updated prediction after learning Exp2	Exp 2	47.12	50.48	+7.13%	6.19%	5.67%

**Table 6:** Sequential prediction accuracy for Rank 1 before and after coupon-based learning.

Prediction Stage	Role in Learning Sequence	Ref. Exp.	Exp. UTS (MPa)	Pred. UTS (MPa)	UTS Error (%)	Full-Curve RMSE (%)	Full-Curve MAE (%)
**—Baseline (no learning)—**							
Predict 0	Blind OOD—no experimental data	Coupon 1	48.21	57.40	**+19.06%**	**21.04%**	**20.67%**
**—After learning Coupon 1—**							
Predict 1	Self-validation after learning Coupon 1	Coupon 1	48.21	48.50	+0.60%	6.93%	6.47%
Predict 1 (blind)	Blind prediction—before learning Coupon 2	Coupon 2	49.21	48.50	−1.44%	8.30%	7.84%
**—After learning Coupon 2—**							
Predict 2	Updated prediction after learning Coupon 2	Coupon 2	49.21	49.48	+0.55%	7.59%	7.16%
Predict 2 (blind)	Blind prediction—before learning Coupon 3	Coupon 3	50.10	49.48	−1.24%	6.51%	6.08%
**—After learning Coupon 3—**							
Predict 3	Updated prediction after learning Coupon 3	Coupon 3	50.10	51.01	+1.83%	6.66%	6.17%

Note: Bold values indicate the initial blind/no-learning baseline error metrics.

**Table 7:** Sequential prediction accuracy for Rank 2 before and after coupon-based learning.

Prediction Stage	Role in Learning Sequence	Ref. Exp.	Exp. UTS (MPa)	Pred. UTS (MPa)	UTS Error (%)	Full-Curve RMSE (%)	Full-Curve MAE (%)
**—Baseline (no learning)—**							
Predict 0	Blind OOD—no experimental data	Coupon 1	50.98	50.88	−0.18%	20.74%	20.25%
**—After learning Coupon 1—**							
Predict 1	Self-validation after learning Coupon 1	Coupon 1	50.98	54.13	+6.18%	7.50%	6.67%
Predict 1 (blind)	Blind prediction—before learning Coupon 2	Coupon 2	50.02	54.13	+8.20%	8.34%	7.45%
**—After learning Coupon 2—**							
Predict 2	Updated prediction after learning Coupon 2	Coupon 2	50.02	51.69	+3.33%	8.21%	7.26%
Predict 2 (blind)	Blind prediction—before learning Coupon 3	Coupon 3	50.98	51.69	+1.39%	8.06%	7.09%
**—After learning Coupon 3—**							
Predict 3	Updated prediction after learning Coupon 3	Coupon 3	50.98	52.58	+3.12%	7.71%	6.99%

**Table 8:** Sequential prediction accuracy for Rank 3 before and after coupon-based learning.

Prediction Stage	Role in Learning Sequence	Ref. Exp.	Exp. UTS (MPa)	Pred. UTS (MPa)	UTS Error (%)	Full-Curve RMSE (%)	Full-Curve MAE (%)
**—Baseline (no learning)—**							
Predict 0	Blind OOD—no experimental data	Coupon 1	47.58	50.23	+5.58%	19.40%	18.65%
**—After learning Coupon 1—**							
Predict 1	Self-validation after learning Coupon 1	Coupon 1	47.58	50.45	+6.04%	7.48%	6.43%
Predict 1 (blind)	Blind prediction—before learning Coupon 2	Coupon 2	47.81	50.45	+5.53%	7.46%	6.45%
**—After learning Coupon 2—**							
Predict 2	Updated prediction after learning Coupon 2	Coupon 2	47.81	49.31	+3.14%	7.74%	6.61%
Predict 2 (blind)	Blind prediction—before learning Coupon 3	Coupon 3	47.20	49.31	+4.48%	7.24%	5.98%
**—After learning Coupon 3—**							
Predict 3	Updated prediction after learning Coupon 3	Coupon 3	47.20	50.07	+6.09%	7.53%	6.13%

## Data Availability

The experimental validation datasets, predicted tensile datasets, software notebook, pseudocode, README file, and technical documentation supporting the findings of this study are available at Zenodo: https://doi.org/10.5281/zenodo.19844820.

## Abbreviations

The following abbreviations are used in this study:

ABS	Acrylonitrile butadiene styrene
AI	Artificial intelligence
ANN	Artificial neural network
ANFIS	Adaptive neuro-fuzzy inference system
DE	Differential evolution
DIC	Digital image correlation
FDM	Fused deposition modeling
FFF	Fused filament fabrication
GA	Genetic algorithm
GBR	Gradient boosting regression
GMDH	Group method of data handling
GP	Gaussian process
GPR	Gaussian process regression
MEX	Material extrusion
MAE	Mean absolute error
ML	Machine learning
OOD	Out-of-distribution
PA12	Polyamide 12
PC	Polycarbonate
PC-ABS	Polycarbonate–acrylonitrile butadiene styrene
PETG	Polyethylene terephthalate glycol-modified
PLA	Polylactic acid
PLA-CF	Carbon-fiber-reinforced polylactic acid
RBF	Radial basis function
RF	Random forest
RMSE	Root mean square error
RoM	Rule of mixtures
RSM	Response surface methodology
SG	Savitzky–Golay
UTM	Universal testing machine
UTS	Ultimate tensile strength
XGBoost	Extreme Gradient Boosting

## References

[1] Goh G.D., Sing S.L., Yeong W.Y. (2021). A review on machine learning in 3D printing: Applications, potential, and challenges. Artif. Intell. Rev..

[2] Nasiri S., Khosravani M.R. (2021). Machine learning in predicting mechanical behavior of additively manufactured parts. J. Mater. Res. Technol..

[3] Nasrin T., Pourkamali-Anaraki F., Peterson A.M. (2024). Application of machine learning in polymer additive manufacturing: A review. J. Polym. Sci..

[4] Tran T.Q., Ng F.L., Kai J.T.Y., Feih S., Nai M.L.S. (2022). Tensile Strength Enhancement of Fused Filament Fabrication Printed Parts: A Review of Process Improvement Approaches and Respective Impact. Addit. Manuf..

[5] Fiberlogy (2025). Easy PET-G. https://fiberlogy.com/en/fiberlogy-filaments/easy-pet-g/.

[6] Fiberlogy PC-ABS Filament. https://fiberlogy.com/en/fiberlogy-filaments/pc-abs-filament/.

[7] Nainaragaram Ramasamy M., Sliva A., Nag A., Ma Q.P., Hilser O., Heliova M., Simha Martynkova G., Brozova S., Dizo J. (2026). Characterization of PC-ABS and PETG Multi-Material Laminates Fabricated by MEX Method. Polymers.

[8] Rayegani F., Onwubolu G.C. (2014). Fused deposition modelling process parameter prediction and optimization using group method for data handling and differential evolution. Int. J. Adv. Manuf. Technol..

[9] Yadav D., Chhabra D., Garg R.K., Ahlawat A., Phogat A. (2020). Optimization of FDM 3D printing process parameters for multi-material using artificial neural network. Mater. Today Proc..

[10] Rajpurohit S.R., Dave H.K. (2020). Prediction and Optimization of Tensile Strength in FDM Based 3D Printing Using ANFIS. Optimization of Manufacturing Processes.

[11] Pazhamannil R.V., Govindan P., Sooraj P. (2021). Prediction of the tensile strength of polylactic acid fused deposition models using artificial neural network technique. Mater. Today Proc..

[12] Manoharan K., Chockalingam K., Ram S.S. (2020). Prediction of tensile strength in fused deposition modeling process using artificial neural network technique. AIP Conf. Proc..

[13] Deshwal S., Kumar A., Chhabra D. (2020). Exercising hybrid statistical tools GA-RSM, GA-ANN and GA-ANFIS to optimize FDM process parameters for tensile strength improvement. CIRP J. Manuf. Sci. Technol..

[14] Tura A.D., Lemu H.G., Mamo H.B., Santhosh A.J. (2023). Prediction of tensile strength in fused deposition modeling process using artificial neural network and fuzzy logic. Prog. Addit. Manuf..

[15] Weake N., Pant M., Sheoran A., Kumar H. (2020). Optimising Parameters of Fused Filament Fabrication Process to Achieve Optimum Tensile Strength Using Artificial Neural Network. Evergreen.

[16] Gao G., Xu F., Xu J., Tang G., Liu Z. (2022). Parametric Optimization of FDM Process for Improving Mechanical Strengths Using Taguchi Method and Response Surface Method. Machines.

[17] Zhang J., Wang P., Gao R.X. (2019). Deep learning-based tensile strength prediction in fused deposition modeling. Comput. Ind..

[18] Chen T., Guestrin C. (2016). XGBoost: A Scalable Tree Boosting System. KDD ’16: Proceedings of the 22nd ACM SIGKDD International Conference on Knowledge Discovery and Data Mining.

[19] Breiman L. (2001). Random Forests. Mach. Learn..

[20] Friedman J.H. (2001). Greedy function approximation: A gradient boosting machine. Ann. Stat..

[21] Cortes C., Vapnik V. (1995). Support-vector networks. Mach. Learn..

[22] Jayasudha M., Elangovan M., Mahdal M., Priyadarshini J. (2022). Accurate Estimation of Tensile Strength of 3D Printed Parts Using Machine Learning Algorithms. Processes.

[23] Nasrin T., Pourali M., Pourkamali-Anaraki F., Peterson A.M. (2023). Active learning for prediction of tensile properties for material extrusion additive manufacturing. Sci. Rep..

[24] Ziadia A., Habibi M., Kelouwani S. (2023). Machine Learning Study of the Effect of Process Parameters on Tensile Strength of FFF PLA and PLA-CF. Eng.

[25] Deb J.B., Chowdhury S., Ali N.M. (2024). An investigation of the ensemble machine learning techniques for predicting mechanical properties of printed parts in additive manufacturing. Decis. Anal. J..

[26] Deka A., Hall J. (2023). Predictive modeling of mechanical properties for fused deposition modeling parts: A focus on processing and environmental parameters. Manuf. Lett..

[27] Liu Z., Mazzei Capote G.A., Grubis E., Pandey A., Blanco Campos J.C., Hegge G.R., Osswald T.A. (2023). Predicting Properties of Fused Filament Fabrication Parts through Sensors and Machine Learning. J. Manuf. Mater. Process..

[28] Patel K.S., Trivedi N., Shah D.B., Joshi S.J. (2024). Prediction of tensile strength using machine learning algorithms in fused deposition modeling. Proc. Inst. Mech. Eng. Part E J. Process Mech. Eng..

[29] Anand S., Satyarthi M.K. (2024). Neural network-based modeling of FFF process for PET-G: Evaluating MLPNN and RBFNN performance in mechanical property prediction. Proc. Inst. Mech. Eng. Part E J. Process Mech. Eng..

[30] Mishra A., Jatti V.S., Sefene E.M., Jatti A.V., Sisay A.D., Khedkar N.K., Salunkhe S., Pagáč M., Abouel Nasr E.S. (2024). Machine learning-assisted pattern recognition algorithms for estimating ultimate tensile strength in fused deposition modelled polylactic acid specimens. Mater. Technol..

[31] Hamouti L., El Farissi O., Outemssa O. (2022). Regression Model for Optimization and Prediction of Tensile Strength of a PLA Prototype Printed. J. Adv. Comput. Intell. Intell. Inform..

[32] Obaidat S., Tamimi M.F., Mumani A., Alkhaleel B. (2024). An artificial neural network-based predictive model for tensile behavior estimation under uncertainty for fused deposition modeling. Rapid Prototyp. J..

[33] Nikzad M.H., Heidari-Rarani M., Rasti R., Sareh P. (2025). Machine learning-driven prediction of tensile strength in 3D-printed PLA parts. Expert Syst. Appl..

[34] Dhankhar G., Satyarthi M.K. (2026). Hybrid ANN-GTO-Based Optimization of Tensile Strength in Layer-by-Layer ABS–PETG Multi-material FDM Composites. J. Mater. Eng. Perform..

[35] Fetecau C., Stan F., Boazu D. (2025). Artificial Neural Network Modeling of Mechanical Properties of 3D-Printed Polyamide 12 and Its Fiber-Reinforced Composites. Polymers.

[36] Özkül M., Kuncan F., Ulkir O. (2025). Predicting Mechanical Properties of FDM-Produced Parts Using Machine Learning Approaches. J. Appl. Polym. Sci..

[37] Seid Ahmed Y., Hassanin H., Nazir A., Khan S. (2025). A machine learning-integrated framework for mechanical property prediction of FDM-printed PLA. Int. J. Adv. Manuf. Technol..

[38] Shunmugesh K., Biju B., Reji A., Rajasekar V., Hiremath S., Anne G., Vishwanatha H.M. (2026). Optimizing 3D printing parameters for enhanced tensile strength and efficiency using machine learning models. Prog. Addit. Manuf..

[39] Irazman H.N.H., Saad M.S., Baharudin M.E., Zakaria M.Z., Nor A.M., Rahim Y.A. (2023). ANN-Based Predictive Modelling for Fused Deposition Modelling: Material Consumption, Tensile Strength and Dimensional Accuracy. Int. J. Eng. Trends Technol..

[40] Al-Wswasi M., Al-Khaleeli W.A., Aufy S.A. (2025). Implement the artificial neural network concept for predicting the mechanical properties of printed polylactic acid parts. Adv. Sci. Technol. Res. J..

[41] Jatti V.S., Tamboli S., Shaikh S., Solke N.S., Gulia V., Jatti V.S., Khedkar N.K., Salunkhe S., Pagáč M., Abouel Nasr E.S. (2024). Optimization of tensile strength in 3D printed PLA parts via meta-heuristic approaches: A comparative study. Front. Mater..

[42] Wei H., Tang L., Qin H., Wang H., Chen C., Li Y., Wang C. (2024). Optimizing FDM 3D printing parameters for improved tensile strength using the Takagi–Sugeno fuzzy neural network. Mater. Today Commun..

[43] Liu Y., Huang W., Wang Z., Zhang J., Liu J. (2025). Machine learning-based mechanical performance prediction and design of lattice structures. Int. J. Mech. Sci..

[44] Qin Q., Wang X., Dai S., Zhong Y., Wei S. (2025). Machine Learning-Based Prediction of Mechanical Properties for Large Bearing Housing Castings. Materials.

[45] Hoang V.H., Tran M.Q., Ngo V.T. (2026). Machine learning-based prediction of the axial load capacity of UHPC strengthened reinforced concrete columns: A comparative analysis. PLoS ONE.

[46] Nga N.T.T. (2026). Data-driven prediction of UHPC compressive strength using a hybrid 1D CNN–GRU network. Res. Eng. Struct. Mater..

[47] Hills M.A., Becker T.H. (2025). Machine Learning-Based Prediction of Fatigue Strength in Additively Manufactured Ti-6Al-4V Parts: A Sensitivity Analysis of Input Features. J. Mater. Eng. Perform..

[48] (2012). Plastics—Determination of Tensile Properties—Part 2: Test Conditions for Moulding and Extrusion Plastics.

[49] PETG/PC-ABS MEX Laminate Characterization Dataset. Zenodo. https://zenodo.org/records/18472960.

